# Perception of quality of an international recreational gymnastics event: the case of the GIMNASTRADA 2019

**DOI:** 10.3389/fspor.2026.1806745

**Published:** 2026-09-28

**Authors:** Vicente Luis-del Campo, Jesús Muñoz-Jiménez, Héctor Valentín Jiménez-Naranjo, Kiko Francisco León

**Affiliations:** 1 Faculty of Sports Sciences, University of Extremadura, Cáceres, Spain; 2 Faculty of Business, Finance and Tourism, University of Extremadura, Cáceres, Spain

**Keywords:** perceived quality, respondents, sporting event, standard, survey instrument

## Abstract

**Introduction:**

The GIMNASTRADA is an annual, non-competitive gymnastics event, held in the city of Cáceres (Extremadura, Spain) since 1996. The 2019 edition was the largest in number of participants with around 3,700 gymnasts and 12,000 spectators. The aim of this study was to determine whether the perception of quality of GIMNASTRADA 2019 was influenced by the type of participation that different groups of attenders developed in this gymnastic event (gymnasts, gymnastics group leaders, family members, audience, organisational staff).

**Methods:**

To this end, a total of 331 participants completed a short four item-questionnaire created ad hoc for this study in order to evaluate the perceived quality of certain management processes existing in this sporting event (e.g., quality of facilities, planning, organisation and development). The questionnaire, based on single-item measures of previous validated scales as EVENTQUAL and IPA4RE, was answered anonymously by the participants using their own mobile devices via a Google Docs form.

**Results:**

The results showed differences in questionnaire scores between groups of participants. It is concluded that the type of participation influenced the perception of quality between groups of attenders in GIMNASTRADA 2019.

**Discussion:**

It would be advisable for sports managers to design sporting events tailored to different participant profiles, including activities adapted to their specific interests, with the aim of providing a positive experience that would promote their participation in future editions.

## Introduction

The GIMNASTRADA is a gymnastic and acrobatic sport event, established in 1996 by the Club Amigos de la Gimnasia de Extremadura (Amigex). It is the second most important gymnastic event in Spain in terms of participation, following the event held in the Canary Islands (International BLUME Festival Gran Canaria). The 23rd edition was held in 2019, exceeding 3,700 registered gymnasts and 12,000 spectators. The event took place on March 30th and 31st, under the motto “For Dreams”. Initially named Gimnastrada Cacereña, it was renamed Gimnastrada Internacional in its fourth edition, and in 2016, it adopted its current name, Gimnastrada Extremadura. In 2026, the 30th edition of this sporting event has been held on March 13 and 14 under the motto “For Us,” involving a total of 3,000 participants and 10,000 spectators approximately. The organizing committee stated that 102 gymnastic choreographies were presented, corresponding to 76 gymnastics organizations. However, for the purpose of this study, data from the 2019 edition were selected because it was the most populous edition in terms of participants and spectators and because it was also the final edition prior to the COVID-19 pandemic.

The organization of this event involves the Amigex club, teachers from the Bachelor’s Degree in Physical Activity and Sport Sciences at the University of Extremadura (UEX), and volunteers, including graduates and students from the aforementioned degree. It also relies on the collaboration of private companies and public institutions such as the University of Extremadura, Cáceres City Council, the Provincial Council of Cáceres, and the Regional Government of Extremadura through its Directorate General of Sports and the Office of Cross-Border Initiatives. The GIMNASTRADA EXTREMADURA event has grown year after year, consolidating itself as the largest sport event in the Autonomous Community of Extremadura and the one with the greatest social impact at the University of Extremadura. Since 2005, the event has been held at the “Ciudad de Cáceres” Multipurpose Pavilion, which has a capacity of 6,500 spectators. According to the organizing committee, 112 gymnastic choreographies were presented in 2019. Specifically, 36 performances came from gymnastics clubs and schools, 13 from cultural and sports associations, 4 from public gymnastics organizations, 16 from private gymnastics organizations, 8 from preschools, 11 from primary schools, 5 from secondary schools, 5 from public or private performing arts centers, 2 from associations for people with disabilities, 3 from adult and senior citizen associations, 1 from a university group at the University of Extremadura (UEX), and 8 from other groups. All the choreographies shared the spirit of promoting and disseminating gymnastic activities, as well as the socio-cultural exchange among the participants, since the choreographies came from the Extremadura region, other regions of Spain, and Portugal.

According to Murphy and Bauman (1), there are three types of Mass Participant Sport Events (MPSEs). The first type of MPSEs would be elite sporting events (e.g., Olympic Games), the second type would be large-scale health promotion events (e.g., city half marathons or fun short-distance runs, recreational triathlons open to amateurs) and the third one would be non-elite mass events with the potential for community-wide participation (e.g., park-based physical activity festivals, community charity walks). We reasoned that the GIMNASTRADA would be a mix of the two last groups of MPSEs because it is a sporting event characterized by: a) promoting physical activity through gymnastics to general public, b) focusing on community to attract participation of diverse population groups, c) developing a less competitive and fun environment and, d) providing social benefits for the host city (e.g., city positioning, opportunity to social interaction). It is important to note that the type of MPSE influenced the participant motivations between non-traditional vs traditional events [e.g., health orientation, personal goal achievement, affiliation; see (2)]. For example, those participants competing in non-traditional events (e.g., obstacle races) reported higher scores on socializing with others, meeting new people or sharing a mutual identity with other participants compared to their peers of traditional events (e.g., half marathons and marathons).

Nowadays, the event success should is evaluated not only through participation numbers or economic incomes but also by the inclusion of participant-centered measures that capture experiential quality (e.g., satisfaction of participating in the event). This variable is increasingly recognized as a primary indicator of event effectiveness on different types of sporting events because it would stimulate future participation, loyalty, word-of-mouth communication, perceptions of service quality, psychological wellbeing, public health outcomes, and economic sustainability. To exemplify, Bernhart et al. (3) found that satisfaction of the psychological needs for autonomy, competence, and relatedness during charity sport events was associated with intentions to participate in future events and maintain physical activity behaviours. Ferreira et al. (4) concluded that participant satisfaction is central to understanding return intentions and evaluating event management performance for sustainable road racing events. Indeed, satisfied participants are more likely to express intentions to re-participate and maintain long-term engagement with a running event (5). Similarly, satisfaction with a running event had a positive impact on the event perception and its atmospheric components [i.e., event organization, relationships between participants, and event place; see (6)]. Also, Lianopoulos et al. (7) stated that participation satisfaction drives positive behavioral intentions among participants of MPSEs and that multiple experiential dimensions contribute to event satisfaction (e.g., affective and relational dimensions of experience showed a positive relation with event satisfaction, while event satisfaction and the relational dimension of experience were associated with event behavioral intentions).

Participation satisfaction is highly relevant in the planning of sporting events, and it should be considered as a key element for organizations focused on sporting events because it constrains the future behavior of those participants (6, 8). Consequently, studying satisfaction (personal state) and perceived quality (comparison between expectations and service delivery) is fundamental for establishing strategies to improve sporting events (9). According to Crespo-Hervás et al. (10), satisfaction is a consequence of service quality and serves as an antecedent to participants’ future intentions (i.e., service quality would be a predictor of satisfaction). However, the perception of satisfaction and quality are subjective concepts, making it crucial that the sporting event be perceived as high-quality by the participant so they can experience pleasure or enjoyment before, during, and/or after the event, increasing their intention to participate in future editions (11). Therefore, sport managers should identify what variables are related to the perceived quality of a sporting event to enhance participantś satisfaction and ensure their loyalty in upcoming editions.

Currently, there are three theoretical positionings that would potentially explain how participation type is expected to influence the experiences and evaluations of participants in sporting events. These frameworks are the Service Quality Theory, Role Theory and Sport Consumer Behavior Theory. The Service Quality Theory is an evaluation method of consumers’ experience of a service (12). It claims that consumers engage with services to obtain benefits that satisfy their needs and expectations. Therefore, the consumers participate in experiential services such as sporting events in different ways, depending on the benefits they seek. For example, Ko and Pastore (13) concluded that sport consumers evaluate sport services differently, scoring higher those dimensions most related to their involvement in the sport setting. Recreational participants may seek enjoyment, health, social interaction. Spectators may prioritize entertainment and emotional stimulation, placing greater emphasis on event atmosphere and crowd experience. Volunteers may focus on organizational support, communication, and recognition. Similarly, Theodorakis et al. (14) found that different user groups evaluate sport services through distinct quality dimensions but depending on their interaction with the service. The Role Theory proposes that individuals’ attitudes, perceptions, behaviors, and expectations are shaped by the social roles they occupy. In this line, participants selected those roles that align with their identities, social relationships and personal goals (15). For example, participants with strong athletic identities are more likely to participate as competitors. Participants with strong fan identities may participate primarily as spectators and community-oriented participants may choose volunteer roles. Therefore, sport identities influenced participation behavior and event involvement (16). Finally, the Sport Consumer Behavior Theory suggests that sport consumers differ in their motivations, levels of involvement, and patterns of consumption, which subsequently influence their perceptions, attitudes, and behaviors. Within this framework, participants engaged with sport according to their psychological attachment, participation motives, and experiential expectations (17). By applying this theory, it would be possible to identify how varying levels of involvement shape spectators’ perceptions of quality, satisfaction, and loyalty within the context of sporting events (18).

Until now, most studies related to attendance at sporting events have been focused on competitive MPSEs and popular sports (14). Thus, the majority of previous studies have investigated the motivation of one type of attenders in sporting events from the perspective of the general public or spectators. For an exception, Písečná and Cihová (19) found differences in the perception of the quality of the event between two groups of respondents (direct participants: athletes, coaches vs indirect participants: family members and friends) that attended to the Slovak Athletics Championships. They reported higher scores in areas such as the event organization, transport and parking in direct participants compared to the indirect ones because these running events are currently conceived more for athletes and coaches rather than for spectators. According to these authors, addressing these novel perspectives would provide useful information to sporting event managers about how to increase spectator attendance. Therefore, the research gap of this study is to measure the perceived quality of a non-competitive sporting event between different types of attenders in GIMNASTRADA 2019: gymnasts, group leaders, family members, spectators and organizers. To date, no previous studies have investigated this influence in recreational sporting events and less popular sports as gymnastics. Specifically, the objective of this study was, from an integrated framework of the Service Quality and Role Theories, to determine whether the perceived quality of GIMNASTRADA 2019 was influenced by the type of participation of attenders in this gymnastic event.

We reasoned that the relationship between the type of participation of attenders in GIMNASTRADA 2019 and their experiences and evaluations in this sporting event can be explained through the complementary frameworks of the Role and Service Quality Theories. Specifically, we argue that the role-specific interactions of participants in this sporting event would shape their perceptions of the gymnastic event. Anchoring these perspectives would provide a robust rationale for understanding why the same sporting event may generate heterogeneous experiences and evaluations across different participant groups. The underlying mechanism was that each participant group encounters different service elements, forming distinct perceptions of service quality, which would subsequently influence their overall event experience, satisfaction, perceived value, and future behavioral intentions (20).

Overall, we expected differences in questionnaire item scores between groups of participants in GIMNASTRADA 2019, as each group was likely to experience the gymnastics event differently depending on their specific role. Therefore, the type of participation of attenders would shape their perceptions and evaluations of the gymnastic event when they were asked about aspects related to facilities, organization, or planning and development of the sporting event. In more detail, we predict that gymnasts may estimate more positive aspects related to quality of the sport facilities compared to other groups of participants. Instead, the groups of leaders and organizers may focus more on organizational processes than other groups; meanwhile, the groups of families and spectators may seek planning and development of the event in comparison with other groups of participants in GIMNASTRADA 2019 (13, 14, 16).

## Methods

### Sample

A convenience sampling method was used, and a total of 331 participants took part in the study, with a mean age of 37.48 years (SD=12.02). Inclusion criteria required in-person attendance at GIMNASTRADA 2019 and voluntary, anonymous completion of the *ad hoc* questionnaire (see next Instrument section for a detail of questionnaire). The sample consisted of 55 gymnasts (M = 27.39; SD = 14.67), 40 group leaders (M = 37.85; SD = 9.97), 182 family members (M = 41.96; SD = 7.29), 39 spectators (M = 34.87; SD = 15.61), and 11 organization members (M = 24.73; SD = 11.21).

The anonymity of all participants was preserved to protect data confidentiality. All respondents were informed about the objectives of the study prior to participation; however, the specific research hypotheses were not disclosed. For minors, parental or legal guardian supervision was required during questionnaire completion. The study adhered to the regulations of the Bioethics and Biosafety Committee of the University of Extremadura.

### Instrument

Currently, different studies have investigated the participants’ perceptions about the service quality in sporting environments. However, this is not the specific case of sporting events because there are few validated scales focused on these type of events (21). The scale more used in the literature to explore attenderś opinions in sporting events has been SERVQUAL, originally composed of 10 dimensions such as tangibles, reliability responsiveness, competence, courtesy, credibility, security, access, communication understanding the customer (22). Other scales included a more reduced number of dimensions; for instance, SPORTSERV was applied to basketball clubs and had five factors [access, reliability, responsiveness, tangibles and security; see (23)]. Similarly, IPA4RE identified five factors high valued by athletes when participated in a 10 km running event [event atmosphere, security, event route, convenience of bib collection and event day information; see (24)]. Also, EVENTQUAL has only four dimensions such as accessibility, personnel, tangibles, and complementary services (21).

As a result, these studies have used different factors to characterize perceived impacts of sporting events (25). Thus, the writing of items pertaining to these factors has been adjusted to each specific study. To amend this shortcoming of the existing literature, we designed *ad hoc* a four-item questionnaire to assess the participants’ perceptions about the service quality in GIMANSTRADA 2019. We reduced the questionnaire length to four questions in order to promote the maximum number of responses from attenders during this gymnastic event as it had to be filled in at the pavilion. Specifically, the items of this study were based on others extracted from EVENTQUAL and IPA4RE because they were previously used to address the spectators satisfaction (passive participants: EVENTQUAL) and amateur runners satisfaction (active participants: IPA4RE) with sporting events. It should be noted that the wording of these four items was adapted for this study to better reflect the reality of the gymnastics event context. These writing modifications were made solely to enhance their readability, clarity, and interpretability, without altering the underlying constructs being assessed.

The four items asked were: i) How was the quality of the sports facility used in GIMNASTRADA 2019 in relation to the location, access, services and safety? (this item was related to items of the venue dimension of EVENTUAL and physical aspects of IPA4RE); ii) How was the quality of the planning and development in GIMNASTRADA 2019 in relation to its dynamism, amenities, parallel activities? (this item was related to items of the event program of EVENTUAL and event experience of IPA4RE); iii) How was the quality of GIMNASTRADA 2019 in relation to its organization (e.g., staff empathy and attention)? (this item was related to items of staff dimension in both EVENTUAL and IPA4RE) and iv) How was the overall quality of the GIMNASTRADA 22019? This last global item assessing the overall quality of the sporting event was included because previous research has shown that overall perceived event quality is an important antecedent of participant satisfaction (10), which subsequently predicts intention to return and destination loyalty (26, 27).

### Variables and design

The independent variable was the type of participation that attenders in GIMNASTRADA developed during the sporting event (Level 1: Gymnasts, Level 2: Group Leaders, Level 3: Family, Level 4: Spectators, Level 5: Organizers). The dependent variable was the perceived quality of management processes (11), in relation to four aspects: sports facility (QUAL_FAC), planning and development (QUAL_PLAN), organization (QUAL_ORG), and overall assessment (QUAL_ALL).

All dependent variables were measured using a 5-point Likert scale, with response options ranging from 1 (very low) to 5 (very High), as previously made in previous studies (6, 19). Specifically, 1 indicated strongly disagree, 2 disagree, 3 neither agree nor disagree, 4 agree, and 5 strongly agree. Consequently, higher scores reflected greater agreement with the statement represented by each item. For example, a mean score of 4 points for the item QUAL_FAC revealed that participants’ perception was agree, reflecting a positive assessment to that aspect of the gymnastic event. The study employed a cross-sectional study design, which was based on questionnaire data collected during GIMNASTRADA 2019. We used this instrument because it provides surveying basic data about the respondents, and it has been the main instrument to collect data from participants of sporting events (28).

### Procedure

The research team designed the questionnaire one month prior to the event. A week before the event, club technical directors were informed via email. During the event, the questionnaire link was distributed via WhatsApp groups and a QR code displayed on the giant screen of the pavilion. The attenders could filled out the questionnaire during the two days that lasted the sporting event (the event took place on 30 and 31 March 2019). The speakers of the event encouraged attenders to respond the questionnaire during breaks of choreographies (see Figure 1).

**Figure 1:**
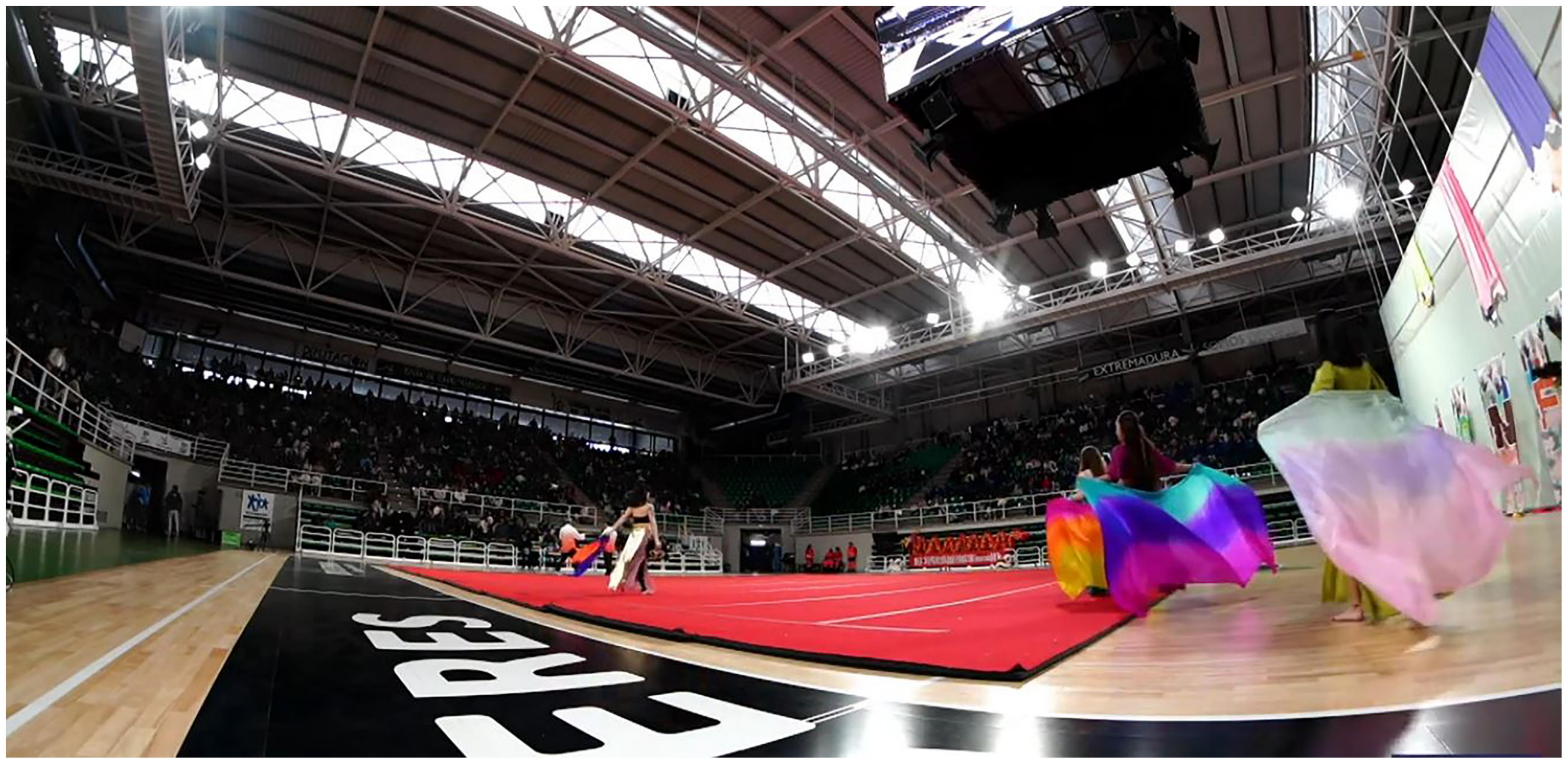
Spectators observing a gymnastic choreography during the GIMNASTRADA.

Participants completed the questionnaire individually using their own electronic devices (e.g., mainly by smartphones, or tablets and laptops). Data was collected using Google Docs and stored in an Excel file for analysis. These responses were subsequently downloaded into an Excel format and subsequently exported to a SPSS file for further statistical analysis.

### Data analysis

The Kolmogorov–Smirnov test indicated a non-normal distribution of the data. Descriptive statistics were calculated, and the Friedman test was used to determine differences in mean ranks among participant groups. *Post-hoc* pairwise comparisons were conducted using the Mann–Whitney U test. This non-parametric test is appropriate for ordinal or ranked data and non-normally distributed variables (29). The magnitude of the differences between pairs of groups was assessed by calculating the effect size (r), using the following formula: r_pb_ = z/√N; where z is the standardized statistic obtained from the Mann–Whitney U test and N is the total sample of respondents of the questionnaire. To interpret the practical value of this effect size, the following convention was used: 0.10: small, 0.30: medium, and 0.50: large (30).

Statistical analyses were conducted using weighted data to account for the unequal number of respondents in each group. Weighting adjusted the contribution of each group of participants to better reflect the target population and to reduce potential bias in parameter estimates arising from unequal group proportions. Analyses were performed at the item level because the aim of the study was to compare the perceived quality between groups of participants in GIMNASTRADA 2019, rather than to validate measurement scales or analyses at the construct level. Accordingly, each statistical comparison corresponded to each of 4 items representing one specific aspect of the perceived quality by respondents in the sporting event. Pairwise Mann–Whitney U tests were adjusted for multiple comparisons using the confirmatory Holm-Bonferroni (HB) procedure, with significance set at an HB-adjusted p < 0.05. All analyses were performed using IBM SPSS Statistics for Windows, Version 25.0 (IBM Corp., Armonk, NY, USA).

## Results

All participant groups showed mean scores above 4 out of 5 for all variables (see Table 1). The organization group was the only one to exceed 4.7 points across all variables. The highest-rated variable across all groups was the Overall Assessment (QUAL_ALL).

**Table 1:** Descriptive statistics (M and SD) by groups of participants.

	QUAL_FAC	QUAL_PLAN	QUAL_ORG	QUAL_ALL
Participant Type	M (SD)	M (SD)	M (SD)	M (SD)
Gymnasts	4.18 (0.92)	4.17 (0.92)	4.19 (0.99)	4.53 (0.56)
Group Leaders	4.53 (0.78)	4.53 (0.84)	4.58 (0.78)	4.85 (0.36)
Family	4.34 (0.87)	4.25 (0.95)	4.27 (0.95)	4.69 (0.53)
Spectators	4.47 (0.76)	4.53 (0.76)	4.65 (0.75)	4.82 (0.38)
Organization	4.82 (0.40)	4.73 (0.46)	4.82 (0.40)	4.82 (0.40)

QUAL_FAC, quality of the sports facility; QUAL_PLAN, planning and development of the sporting event; QUAL_ORG, organization of the sporting event; QUAL_ALL, overall assessment of the sporting event; M, mean values; SD, standard deviation.

Mean ranks indicated that gymnasts group had the lowest scores in all variables, followed by family members, compared to the other groups of participants. Conversely, the organization group showed the highest mean ranks, except for QUAL_ALL, where group leaders scored the highest values than the other groups (see Table 2).

**Table 2:** Mean rank (MR) by groups of participants.

	QUAL_FAC	QUAL_PLAN	QUAL_ORG	QUAL_ALL
Participant Type	MR	MR	MR	MR
Gymnasts	504.56	510.02	504.07	479.68
Group Leaders	625.38	644.43	633.76	637.04
Family	556.88	548.16	532.13	560.16
Spectators	596.89	632.37	663.85	622.46
Organization	718.95	685.36	697.77	621.14

MR, mean rank; QUAL_FAC, quality of the sports facility; QUAL_PLAN, planning and development of the sporting event; QUAL_ORG, organization of the sporting event; QUAL_ALL, overall assessment of the sporting event; MR, mean rank.

The Kruskal–Wallis test confirmed significant differences among types of participants for all variables (H = 28.13 in QUAL_FAC; H = 33.14 in QUAL_PLAN; H = 57.09 in QUAL_ORG; y H = 31.83 in QUAL_ALL; *p*  < 0.001 for all variables). Specifically, pairwise comparisons in the QUAL_FAC variable showed that the organization group achieved significantly higher scores than families and spectators (with medium-to-large effect sizes), and gymnasts (with a large effect size). Also, the group of gymnasts reported lower values than leaders (with a medium effect size).

For QUAL_PLAN and QUAL_ORG variables, the groups of gymnasts and families reported significantly lower scores compared to leaders, spectators and organizers (with small-to-medium effect sizes). Again, the gymnasts displayed lower values than leaders, spectators and organizers for the QUAL_ALL variable (with medium effect sizes). Thus, families showed lower scores compared to leaders and spectators (with small-to-medium effect sizes) (See Table 3).

**Table 3:** Pairwise comparisons between groups of attenders in GIMNASTRADA 2019 for all dependent variables

Participant Type	Quality of the sports facility (QUAL_FAC)					
	MR(a)	MR(b)	U	HB-p	z	r
G1(a) vs G2(b)	75.95	93.53	2637	0.07*	2.50	0.26
G1(a) vs G3(b)	354.84	390.53	17976	0.60	1.27	0.08
G1(a) vs G4(b)	107.64	127.45	4380	0.15	2.09	0.22
G1(a) vs G5(b)	48.95	71.05	1152	0.00*	4.18	0.51
G2(a) vs G3(b)	462.90	413.43	38112	0.09	2.34	0.16
G2(a) vs G4(b)	160.38	152.42	10815	1	0.90	0.10
G2(a) vs G5(b)	88.55	102.50	3366	0.11	2.21	0.31
G3(a) vs G4(b)	448.86	480.66	63620	0.28	1.67	0.11
G3(a) vs G5(b)	384.18	495.14	17052	0.00*	4.31	0.31
G4(a) vs G5(b)	121.39	148.95	4920	0.01*	3.17	0.45

Participant Type	Planning and development of the sporting event (QUAL_PLAN)					
	MR(a)	MR(b)	U	BH-p	z	r
G1(a) vs G2(b)	73.78	93.68	2499	0.02*	2.85	0.29
G1(a) vs G3(b)	366.24	391.23	18292	1	0.87	0.06
G1(a) vs G4(b)	102.13	128.29	4030	0.02*	2.82	0.29
G1(a) vs G5(b)	49.89	69.18	1209	0.00*	3.50	0.43
G2(a) vs G3(b)	481.80	412.67	36324	0.01*	3.23	0.22
G2(a) vs G4(b)	157.88	154	11115	1	0.46	0.05
G2(a) vs G5(b)	91.85	96.50	3762	1	0.72	0.10
G3(a) vs G4(b)	443.66	510.24	58760	0.01*	3.48	0.23
G3(a) vs G5(b)	387.54	482.77	18132	0.00*	3.62	0.26
G4(a) vs G5(b)	125.66	136.68	5730	0.99	1.29	0.18

Participant Type	Organization of the sporting event (QUAL_ORG)					
	MR(a)	MR(b)	U	BH-p	Z	r
G1(a) vs G2(b)	74	93.58	2511	0.02*	2.87	0.29
G1(a) vs G3(b)	367.72	386.84	18372	1	0.68	0.04
G1(a) vs G4(b)	94.54	127.43	3620	0.00*	4.03	0.42
G1(a) vs G5(b)	49.06	69.86	1164	0.00*	3.50	0.43
G2(a) vs G3(b)	481.85	407.88	35358	0.00*	3.51	0.24
G2(a) vs G4(b)	148.06	156.20	10507.5	0.81	1.10	0.12
G2(a) vs G5(b)	90.20	99.50	3564	0.37	1.54	0.22
G3(a) vs G4(b)	429.87	532.78	51100	0.00*	5.51	0.37
G3(a) vs G5(b)	381.71	497.68	16620	0.00*	4.50	0.32
G4(a) vs G5(b)	124.78	129.41	5880	1	0.65	0.09

Participant Type	Overall assessment of the sporting event (QUAL_ALL)					
	MR(a)	MR(b)	U	BH-p	z	r
G1(a) vs G2(b)	69.92	93.42	2290.50	0.00*	3.90	0.40
G1(a) vs G3(b)	337.72	391.23	16752	0.13	2.14	0.14
G1(a) vs G4(b)	100.65	131.74	3950	0.00*	3.83	0.40
G1(a) vs G5(b)	52.33	67.18	1341	0.02*	2.95	0.36
G2(a) vs G3(b)	466.85	411.23	36522	0.02*	3.01	0.20
G2(a) vs G4(b)	159	155	11115	1	0.58	0.07
G2(a) vs G5(b)	92.92	90.36	3753	1	0.49	0.07
G3(a) vs G4(b)	447.49	496.82	62630	0.01*	3.03	0.20
G3(a) vs G5(b)	390.02	431.50	21252	0.20	1.83	0.13
G4(a) vs G5(b)	131.08	130.77	6420	1	0.43	0.06

G1 = Gymnasts; G2 = Group Leaders; G3 = Family; G4 = Spectators; G5 = Organization; MR = Mean Rank; a= group 1 from pairwise comparisons; b = group 2 from pairwise comparisons; U = Mann Whitney test; HB-p = Holm-Bonferroni adjusted p-values; z = standardized test statistics from each Mann–Whitney U test; r = effect size;

* p < 0.05.

## Discussion

This study addressed how the type of participation of attenders in GIMNASTRADA 2019 influenced their perceived quality of the sporting event. The rationale used to interpret results was the Role and Service Quality Theories because they are suitable frameworks to provide the source of differences (i.e., participants’ social roles) but also the mechanism through which those differences affect outcomes (i.e., perceived service quality).

The results confirm that the type of participation influenced the perceived quality of GIMNASTRADA 2019, specifically regarding management aspects such as planning, development, and organization. Regardless of the type of participation, participants in the sporting event score values higher than 4 out of 5 points in all study variables. Based on these data, it can be concluded that the participants, independently of the group they belonged to, have a positive perception of the quality of the sporting event. Specifically, they point high scores to the questions of the questionnaire because they are satisfied with GIMNASTRADA 2019. In this regard, satisfaction with the event is a variable related to the intention to revisit and recommend the event to others (31). We also believe that the sporting event provides participants with rewarding emotions and experiences during the gymnastics performances, and also during breaks between shows, thanks to the entertainment provided by clowns and group dances. These sensory experiences create unique opportunities for enjoyment and could help foster participant loyalty in future editions of the event (32).

A more detailed analysis of the results shows that there is a differentiated perception of quality of GIMNASTRADA 2019 between the different groups of participants. These findings emphasize that sporting events are multi-dimensional service ecosystems where the satisfaction of groups participation is shaped by role-specific value perceptions (33). In this line, Písečná and Cihová (19) have recently found differences in the perception of the quality of the event between direct participants (athletes, coaches) and indirect participants (family members and friends). However, the higher values in that study are found for the direct participants compared to the indirect ones, suggesting that the organization of the event is more focused on athletes and coaches, meanwhile the experience of family members and friends is less relevant. Contrary, the group of gymnasts in our study have the lowest quality perception of the sporting event than leaders, spectators, and organizers in QUAL_PLAN, QUAL_ORG, and QUAL_ALL. Also, they showed lower scores in QUAL_FAC when compared to leaders. Several reasons could explain these different perceptions of quality among the groups of participants.

First, active participants as gymnast are a demanding group who require different services and activities (34). For example, accommodation facilities and activities are important aspects for sports tourists compared to non-sports tourists (35). Also, organizational aspects are important to active sports tourists because they travel to participate and expect the organizational details to function properly (36, 37). Similarly, active sports tourists participating in several marathons in Germany show a strong relationship between physical and organizational aspects of the sporting event. In contrast, passive sports tourists show a stronger association between emotional and social aspects (38). Additionally, race participants expect to have good signage and control during the event (e.g., rules and safety) but also other variables seem to contribute to build a positive atmosphere of the sporting event [e.g., the attractiveness and difficulty of the running route, the willingness to return of the relation to other participants; see (6)]. Therefore, any errors or deficiencies in the management of these services observed by the gymnasts during the event result in lower scores on the questionnaire items. Thus, we argue that gymnasts may have some pressure to perform the gymnastic choreography because they trained for a long time although the gymnastic event was not competitive. If some error occurs during performances, this may negatively affect their perception of the quality of the sporting event.

Second, spectators show higher scores than gymnasts in QUAL_PLAN, QUAL_ORG, and QUAL_ALL variables because their satisfaction would be closely linked to emotional and sensory experiences, which is aligned with the fun atmosphere driven by the recreational character of the gymnastic event. We reasoned that these higher scores in spectators are due to their emotional experiences gained during the sporting event. Specifically, they benefit from this type of experiences when interacted with other people seated in the stands of the pavilion or when interacting with the speakers and/or clowns to perform some spontaneous performances between choreographies, reinforcing social ties between individuals that generally appear during sporting events (39). For example, attending a sporting event as a spectator creates positive social effects, such as belonging to a wider community (e.g., interacting with people who share the same interest in gymnastics) or having a closer sense of belonging to that community (40). Similarly, spectators reported higher values compared to families in the aforehand variables because they evaluated the sporting event primarily through experiential and hedonic dimensions, including entertainment value, atmosphere, and engagement (41). We reasoned that families would be also more sensitivity to service delivery conditions (e.g., accessibility, comfort, safety, and other supporting services). From this perspective, family attendees would assume a dual-role condition combining emotional engagement and some logistical responsibility. This would increase their sensitivity to organizational shortcomings such as inadequate seating arrangements, insufficient family-oriented facilities, congestion, and time inefficiencies as occurred with gymnasts. Empirical research has shown that families accompanying athletes report lower satisfaction when events are not explicitly designed to accommodate their specific needs (19, 42).

Third, we suggest that the group leaders and organizers of the event show a higher quality perception of the sporting event than gymnasts (for QUAL_PLAN, QUAL_ORG and QUAL_ALL) and families (for QUAL_PLAN, QUAL_ORG) because the articulation of their image of the sporting event was more focused on aspects of planning and development (e.g., coordination, access, safety and control of the gymnasts during the event, as well as the arrangement of space and materials for the normal development of the gymnastic performances). For example, organizers in planned events tend to often contextualize limitations within organizational constraints, resulting in comparatively more favorable assessments (43). In contrast, the gymnasts’ perception of the event may have been more closely associated with their physical experience during the event (i.e., the physical effort exerted during the performance), their social experience (i.e., their interaction with other gymnasts), and their relationship with the environment [i.e., their interaction with the sports facility—the pavilion—and more specifically with the gymnastics hall where the performances took place; see (38)]. This more complex association in shaping the perception of the sporting event could explain the lower scores of gymnasts compared to the other groups. Altogether, gymnasts and family members experience a larger “expectation–performance gap” than the other groups of participants, which originates from the SERVQUAL model of service quality (22), leading to a lower perceived service quality of the gymnastic event because they have high expectations of the sporting event.

Beyond the differences between groups of attenders, organizers should understand the interests of their participants at the destination, as well as the service options offered by the event, to foster a positive relationship between destination and event. This increases participants’ likelihood of returning to the sporting event in future editions (44). In this line, sports event organizers should carefully manage the image of the event projects among participants, highlighting emotional, physical, and organizational aspects for active participants, or the social and historical aspects of the destination city for passive participants (38). In this line, Jiménez-Galán et al. (45) state that coaches, instructors, and staff who work daily with gymnasts should design specific training programs to increase the gymnasts’ enjoyment during artistic gymnastic performances. Furthermore, sports event organizers should reframe the design of the sporting event towards the joint management of quality and enjoyment, as the latter influences the hedonistic satisfaction of participants, positively impacting their happiness and their future intention to attend the event again (46).

### Limitations, strengths and future perspectives

The primary limitation is the small sample size, which limits the generalizability of its results to the total population that attended GIMNASTRADA 2019. A sample size of 387 participants would be required to ensure external validity in this study, calculated using the Yamane formula: *n* = N/[1+N(e²)], where n represents the required sample size, N is the total population (12,000 spectators), and e is the desired margin of error (5%). We were only able to recruit 331 respondents, even though the questionnaire was short and quick to complete. In addition, an *a priori* power analysis conducted with G*Power (version 3.1.9.7) indicates that 53 participants per group are needed to achieve 80% statistical power, an alpha level of .05, and a medium effect size (d = 0.5), using the Mann–Whitney U test (47). In this study, the groups of gymnasts (*n* = 55) and families (*n* = 181) reached this minimum number of participants by group (*n* = 53), but not the groups of leaders (*n* = 40), spectators (*n* = 39) and organizers (*n* = 11). Therefore, future data collection should involve volunteers with portable devices to facilitate higher rate of responses between groups of participants *in situ*. Also, the unequal group sizes would be recognized as a study limitation, and the results should be interpreted with caution to avoid misunderstandings.

The strength of this study is that the original empirical findings are interpreted within the lens of the Role and Service Quality Theories. This integrated rationale provides novel explanations about how different participant groups (e.g., gymnasts, leaders, families, spectators, organizers) evaluated this gymnastic event based on their distinct roles and expectations, and how the gap between their expected and experienced event services influence their perceived service quality and satisfaction with the sporting event. Further statistical analyses could be planned in order to classify the groups of participants differently; for instance, by direct participants (athletes, coaches) and indirect participants (family members and friends), as recently made by (19). Similarly, future studies could integrate into the same research project a comparison between active participants with different roles (e.g., athletes, organizers) and passive participants (families and spectators) to address whether the level of involvement of the participants in the sporting event would influence their quality perception as previous studies have addressed separately but not collectively (for an exception; see (11)).

Additionally, employing a mixed-methods approach (i.e., using quantitative and qualitative methodologies) would enrich data interpretation. For example, using not only one instrument as the questionnaire but also some type of survey to deepen into the opinions given by the attenders of the sporting event. Next editions of the gymnastic event should explore experiential and sensory marketing to enhance its value and analyze perceived quality longitudinally (before, during, and after the event).

### Practical applications

Currently, there are great number and diversity of sporting events, creating a highly competitive environment in which sport managers must strategically differentiate their events to remain attractive for potential participants. In this context, identifying the preferences, motivations, and expectations of event participants is critical for achieving advantage in the sport event marketplace. Therefore, a better understanding of the motivational profiles of event participants provides sport managers a better event planning, maximizing the benefits derived from their event participation (2). Specifically, identifying the underlying motives that drive participants to attend events can facilitate the development of targeted marketing strategies, tailored event experiences, and effective engagement initiatives.

A clarification regarding participantś motivations may contribute to enhance their attendee satisfaction, fostering long-term loyalty, and increasing the likelihood of repeat participation. It is important to note that satisfied participants develop stronger emotional attachment and commitment toward sporting events, increasing the likelihood of repeat participation. For example, they may improve logistical aspects for gymnasts, such as the number of changing rooms and warm-up areas. For group leaders, specific coordination meetings could resolve scheduling and access issues. For the public and families, the experience could be intensified through interactive activities and contests during intervals. Also, the event should be leveraged to build social capital in the local community, investing in equipment and promoting gymnastics as an inclusive, recreational practice.

Finally, sports event organizers should strengthen service quality management, as it is an indicator of participants’ behavioral intentions (48). For example, if the sport event is well-managed and runner satisfaction is high, this positive perception of the event could influence the sports tourist’s likelihood of returning to the destination (6, 27). Similarly, the willingness to return among sports tourists would be high if the perceived service quality and satisfaction levels are high (49, 50). In this vein, GIMNASTRADA should reinforce not only recreational purposes but also the promotion of physical activity and public health. Participant satisfaction plays a critical role in achieving these objectives because positive experiences encourage individuals to maintain active lifestyles. Therefore, participation satisfaction should be treated as a key outcome and strategic planning variable in sporting events. Altogether, the design of this gymnastic event from this perspective of satisfying the needs of their different groups of participants would strengthen their long-duration sustainability (51).

## Data Availability

The raw data supporting the conclusions of this article will be made available by the authors, without undue reservation.
